# Experimental Research on Stability and Natural Convection of TiO_2_-Water Nanofluid in Enclosures with Different Rotation Angles

**DOI:** 10.1186/s11671-017-2170-1

**Published:** 2017-06-08

**Authors:** Cong Qi, Guiqing Wang, Yifeng Ma, Leixin Guo

**Affiliations:** School of Electrical and Power Engineering, China University of Mining and Technology, 221116 Xuzhou, China

**Keywords:** Natural convection, Nanofluid, Rotation angle, Stability

## Abstract

The stability and natural convection heat transfer characteristics of TiO_2_-water nanofluid in enclosures with different rotation angles (*α* = −45°, *α* = 0°, *α* = 45°, and *α* = 90°) are experimentally investigated. The effects of different pH values and doses (*m*) of dispersant agent on the stability of TiO_2_-water nanofluid are investigated. It is found that TiO_2_-water nanofluid with *m* = 6 wt% and pH = 8 has the lowest transmittance and has the best stability. The effects of different rotation angles (*α* = −45°, *α* = 0°, *α* = 45°, and *α* = 90°), nanoparticle mass fractions (wt% = 0.1%, wt% = 0.3%, and wt% = 0.5%) and heating powers (*Q* = 1 W, *Q* = 5 W, *Q* = 10 W, *Q* = 15 W, and *Q* = 20 W) on the natural convection heat transfer characteristics are also studied. It is found that the enclosure with rotation angle α = 0° has the highest Nusselt number, followed by the enclosure with rotation angles *α* = 45° and *α* = 90°, the enclosure with rotation angle *α* = −45° has the lowest Nusselt number. It is also found that natural convection heat transfer performance increases with the nanoparticle mass fraction and heating power, but the enhancement ratio decreases with the heating power.

## Background

Since nanofluid is prepared, due to its excellent heat conducting properties [1–3], nanofluid is widely applied in heat transfer field [4–6], especially in the natural convection field [7–9].

Natural convection heat transfer characteristics of nanofluid are numerically investigated by many researchers. He et al. [10, 11] applied a single-phase and a two-phase lattice Boltzmann methods to numerically study the natural convection heat transfer of Al_2_O_3_-water nanofluid in a square cavity, respectively. Sheikholeslami et al. [12] investigated the magnetohydrodynamic natural convection heat transfer characteristics of a horizontal cylindrical enclosure with an inner triangular cylinder filled with Al_2_O_3_-water nanofluid by a lattice Boltzmann simulation method. Uddin et al. [13] studied the natural convection heat transfer of various nanofluids along a vertical plate embedded in porous medium based on the Darcy-Forchheimer model. Meng et al. [14] numerically investigated the natural convection of a horizontal cylinder filled with Al_2_O_3_-water nanofluid. Ahmed et al. [15] used a two-phase lattice Boltzmann method to study the natural convection of CuO-water nanofluid in an inclined enclosure. Qi et al. [16] numerically simulated the natural convection of Cu-Ga nanofluid in an enclosure.

In addition to above numerical simulations on the natural convection of nanofluid, the experimental studies on natural convection of nanofluid are done by more and more researchers. Li et al. [17] experimentally investigated the natural convection heat transfer of ZnO-EG/water nanofluid. Hu et al. [18, 19] experimentally studied the natural convection heat transfer enhancement of a square enclosure filled with TiO_2_-water and Al_2_O_3_-water nanofluids respectively. Ho et al. [20] experimentally studied the natural convection heat transfer of vertical square enclosures with different sizes filled with Al_2_O_3_-water nanofluid. Heris et al. [21–23] experimentally investigated the convective heat transfer characteristics of different kinds of nanofluid (Cu/water, Al_2_O_3_-water, and CuO-water) in circular tubes, respectively. Mansour et al. [24] experimentally investigated the mixed convection of an inclined tube filled with Al_2_O_3_-water nanofluid. Chang et al. [25] experimentally investigated the natural convection of Al_2_O_3_-water nanofluid in thin enclosures. Wen et al. [26, 27] experimentally investigated the convective heat transfer characteristics of Al_2_O_3_-water nanofluids and TiO_2_-water nanofluids under laminar flow conditions, respectively. Xuan et al. [28] experimentally studied the convection heat transfer of Cu-water nanofluid in a straight brass tube.

Above literatures made a great contribution in the natural convection heat transfer characteristics of nanofluid. However, the natural convection heat transfer enhancement of enclosures with different rotation angles filled with nanofluid is needed to be investigated further. Hence, the stability and natural convection heat transfer characteristics of TiO_2_-water nanofluid in enclosures with different rotation angles (*α* = −45°, *α* = 0°, *α* = 45°, and *α* = 90°) are experimentally investigated in this paper.

## Method

### Preparation of Nanofluid and its Stability

**Fig. 1:**
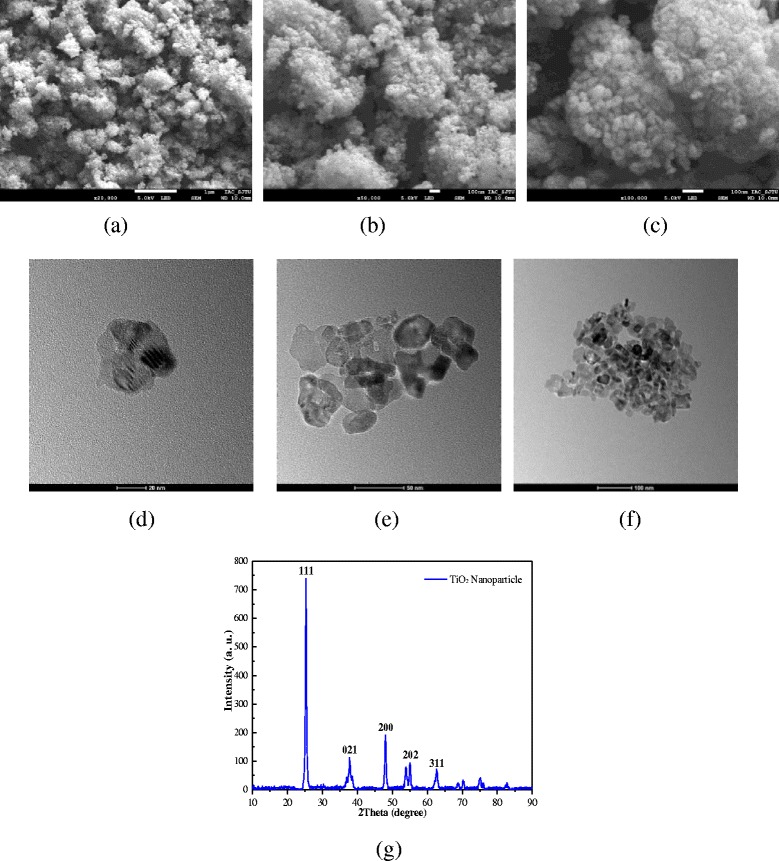
Morphology of nanoparticles. SEM, TEM, and XRD images of TiO_2_ nanoparticles at different magnification times. **a** SEM × 20000. **b** SEM × 50000. **c** SEM × 100000. **d** TEM 20 nm. **e** TEM 50 nm. **f** TEM 100 nm. **g** XRD

TiO_2_ is chosen as the nanoparticles. Figure 1 presents the SEM, TEM, and XRD images of TiO_2_ nanoparticles at different magnification times. It can be found that from SEM images that the nanoparticles easily gather together, and it is necessary to take some measures to prepare the stable nanofluids. It can be also found that from TEM images that the particle size is about 10 nm, and the shapes of nanoparticles are flat. Flat nanoparticles have a larger heat transfer area than spherical nanoparticles at the same mass fraction, which is advantageous to heat transfer enhancement. Figure 1g shows the XRD patterns of the TTP-A10 TiO_2_ nanoparticle. As observed, the strong and sharp peaks suggest that the TTP-A10 TiO_2_ nanoparticle sample is highly crystalline. The average particle size of the sample can be calculated by the Scherrer equation presented in Eq. (1). The TiO_2_ nanoparticle sizes are 6, 9, 14, 20, and 35 nm calculated by these diffraction peak values (111, 200, 021, 202, and 311), and the smallest nanoparticle sizes are about 6 and 9 nm based on the diffraction peak values (111 and 200). The big nanoparticle sizes may be caused by the aggregation of nanoparticles. The smallest values (6 and 9 nm) may be the real sizes of nanoparticle, the size of a few nanoparticles may be 6 nm, and most nanoparticle sizes may be about 9 nm, which are more close to the description supplied by the manufacturer (10 nm) and the TEM images (10 nm).

1 $$ {D}_{\mathrm{c}}=\frac{k\lambda}{\beta \cdot \cos \theta} $$

where *k* is the value for the shape factor, and *k* = 0.9; *λ* is the X-ray wavelength; and *β* is the line broadening full width at half maximum (FWHM) of peak height in radians, and *θ* is the Bragg diffraction angle.

**Fig. 2:**
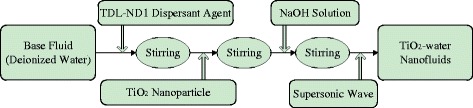
Preparation of nanofluids. Preparation procedure of TiO_2_-water nanofluids by a two-step method

**Fig. 3:**
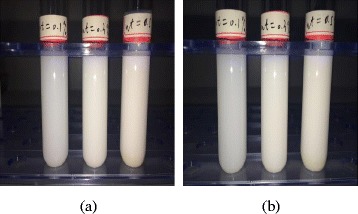
Stability observation of TiO_2_-water nanofluid. TiO_2_-water nanofluid at different times. **a** Before laying. **b** After 72 h

TiO_2_-water nanofluid with different nanoparticle mass fractions (wt% = 0.1%, wt% = 0.3%, and wt% = 0.5%) is prepared by the two-step method, which is shown in Fig. 2. Mechanical stirring time is half an hour for each of the sub-steps, and the sonication time is 40 min. Table 1 shows the information of some materials and equipments in the preparation of nanofluids. Figure 3 shows the TiO_2_-water nanofluid before laying and after 72 h. It can be seen that there is little deposition of nanoparticles in the test tube and nanofluid prepared in this paper shows a good stability.

**Table 1:** Information of materials and equipments. Information of some materials and equipments in the preparation of nanofluids

Materials and equipments	Manufacturer	Properties
TiO_2_ nanoparticles	Nanjing Tansail Advanced Materials Co., Ltd.	Type: TTP-A10; Crystal form: anatase; Particle diameter:10 nm
Base fluid (deionized water)	Prepared by ultrapure water device	Resistivity: 16–18.2 MΩ•cm@25 °C
Ultrapure water device	Nanjing Yeap Esselte Technology Development Co., Ltd.	Type: EPED-E2-10TJ
Dispersant agent	Nanjing Tansail Advanced Materials Co., Ltd.	Type: TDL-ND1; Element: macromolecule polymers; Scope of application: water or solvent (base fluid)
Ultrasonic oscillation device	Shenzhen Jeken Ultrasonic Technology Co., Ltd.	Type: PS-100A; Ultrasonic frequency: 40,000 HZ
Magnetic stirring apparatus	Shanghai MeiYingPu Instrument Manufacturing Co., Ltd.	Type: MYP11-2 Rotate speed: 50 ~ 1500 r/min
Electronic analytical balance	Shanghai Hengping Instrument and Meter Factory	Type: FA2204; Precision: 0.1 mg

**Fig. 4:**
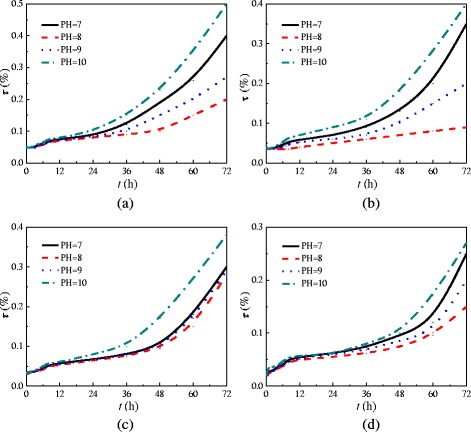
Transmittance of TiO_2_-water nanofluid. Transmittance (*τ*) changes of TiO_2_-water nanofluid (wt% = 0.5%) under different pH values with times (*h*) at different doses (*m*) of dispersant agent. **a** *m* = 5 wt%. **b** *m* = 6 wt%. **c** *m* = 7 wt%. **d** *m* = 8 wt%

In addition to the study on whether there is deposition of nanoparticles in the test tube, the effects of transmittance (*τ*) of nanofluid on its stability are also discussed. Figure 4 gives the transmittance (*τ*) changes of TiO_2_-water nanofluid (wt% = 0.5%) with different pH values and doses (*m*) of dispersant agent. The transmittance is measured by an ultra violet visible spectrophotometer (UV-1800(PC)). As we know, if the nanoparticles uniformly distribute in the water, the nanoparticles will reflect the most light and have a high reflectance (a low transmittance). Hence, the stability of nanofluid is inversely proportional to the transmittance, and the stable nanofluid has a low transmittance. It can be found from Fig. 4 that the nanofluid with *m* = 6 wt% and pH = 8 has the lowest transmittance and has the best stability. The nanofluids with different nanoparticle mass fractions in this experiment are prepared at *m* = 6 wt% and pH = 8, which can ensure the stability of nanofluids.

### Experimental System

**Fig. 5:**
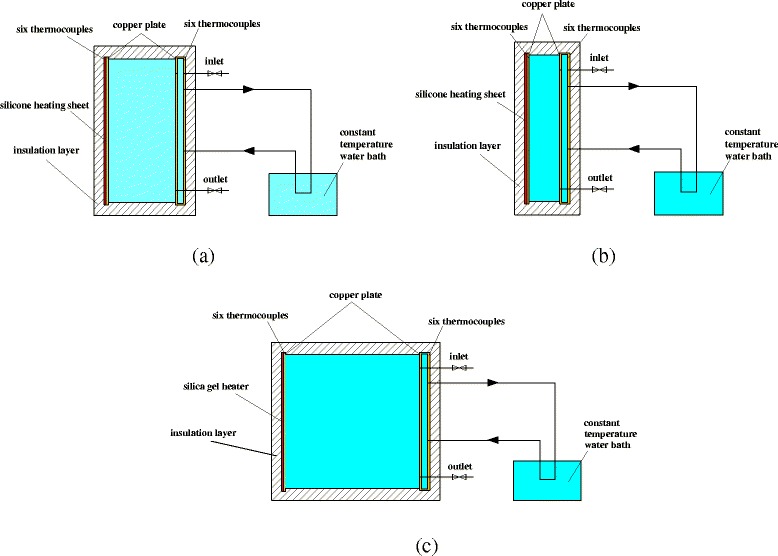
Schematic diagrams of experimental sets. Schematic diagrams of three different aspect ratio experimental sets. **a** *A* = 1:2. **b** *A* = 1:4. **c** *A* = 1:1

Figure 5 shows the schematic diagrams of the three experimental sets. The sizes of the three rectangular enclosures are 10 cm (width) × 20 cm (height), 5 cm (width) × 20 cm (height), and 20 cm (width) × 20 cm (height). The width and height are defined as *W* and *H*, respectively, and the aspect ratio (*A*) of the enclosure is defined as *A = W /H*. The left wall (copper plate) of the enclosure is heated by a silicone heating sheet connected to a DC power. The right wall (copper plate) of the enclosure is cooled by the cooling water in a small cavity (the material is also copper) connected to a constant temperature water bath. The temperatures of two sides of the enclosure are obtained by six thermocouples connected to a data acquisition instrument (Agilent 34972A). The outside insulation layer is used to prevent the heat losing.

**Fig. 6:**
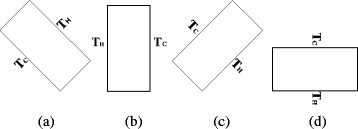
Schematic diagram of rotation angles. Schematic diagram of the enclosures with four different rotation angles. **a** α = −45°. **b** α = 0°. **c** α = 45°. **d** α = 90°

The natural convection heat transfer characteristics of the two enclosures with different rotation angles (*α* = −45°, *α* = 0°, *α* = 45°, and *α* = 90°) filled with TiO_2_-water nanofluid are investigated in this paper. For the enclosure with *α* = −90°, the top wall is the hot wall and the bottom wall is the cold wall, and the heat transfer in the enclosure is mainly heat conduction. However, the manuscript mainly investigates the natural convection heat transfer of nanofluid in the enclosure, hence, the enclosure with *α* = −90° is not considered in this manuscript. Figure 6 shows the schematic diagram of enclosures with different rotation angles.

### Data Processing

The power *Q* provided by the silicone heating sheet is as follows:

2 $$ Q=\mathrm{U}\mathrm{I} $$

where *U* and *I* are the voltage and electricity of the DC power respectively.

The effective power *Q*_net_ is as follows:

3 $$ {Q}_{\mathrm{net}}= Q-{Q}_{\mathrm{loss}} $$

where *Q*_loss_ is the heat loss measured by a heat flow meter.

The temperature of copper plate side next to silicone heating sheet ${T}_{\mathrm{H}}^{*}$ is as follows:

4 $$ {T}_{\mathrm{H}}^{*}=\frac{\left({T}_1+{T}_2+\cdot \cdot \cdot +{T}_6\right)}{6} $$

where *T*_1_, *T*_2_, …, *T*_6_ are the temperatures of thermocouples.

The temperature of copper plate side (left side of enclosure) next to nanofluid *T*_H_ is as follows:

5 $$ {T}_{\mathrm{H}}={T_{\mathrm{H}}}^{*}-\frac{Q_{\mathrm{net}}\delta}{A{\lambda}_{\mathrm{w}}} $$

where *δ* = 0.005m is the thickness of the copper plate, *A* is the area of the copper plate, *λ*_*w*_is the thermal conductivity of the copper plate.

The temperature of copper plate side (right side of enclosure) next to insulation layer *T*_C_^∗^ is as follows:

6 $$ {T}_{\mathrm{C}}^{*}=\frac{\left({T}_7+{T}_8+\cdot \cdot \cdot +{T}_{12}\right)}{6} $$

where *T*_7_, *T*_8_, …, *T*_12_ are the temperatures of thermocouples in the right side of the enclosure.

When the thermal equilibrium state is reached, the temperature of cooling water is the same with the temperature of the copper plate side next to the cooling water. The temperature of the copper plate side (right side of enclosure) next to nanofluid *T*_C_ can be calculated as follows:

7 $$ {T}_{\mathrm{C}}={T_{\mathrm{C}}}^{\ast }-\frac{2{Q}_{\mathrm{net}}\delta}{A{\lambda}_w} $$

The qualitative temperature *T*_m_ is defined as follows:

8 $$ {T}_{\mathrm{m}}=\frac{T_{\mathrm{H}}+{T}_{\mathrm{C}}}{2} $$

The convective heat transfer coefficient *h* is as follows:

9 $$ h=\frac{Q_{\mathrm{net}}}{A\left({T}_{\mathrm{H}}\hbox{-} {T}_{\mathrm{C}}\right)} $$

Nusselt number is defined as follows:

10 $$ \mathrm{Nu}=\frac{h\cdot W}{\lambda_{\mathrm{f}}} $$

where *λ*_f_ is the thermal conductivity of the fluid in the enclosure.

### Uncertainty Analysis

The error transfer formula of the convective heat transfer coefficient is as follows [19]:

11 $$ \begin{array}{l}\frac{\varDelta h}{h}=\left|\frac{\partial \ln h}{\partial {Q}_{net}}\right|\varDelta {Q}_{{}_{net}}+\left|\frac{\partial \ln h}{\partial A}\right|\varDelta A+\left|\frac{\partial \ln h}{\partial \left({T}_{\mathrm{H}}-{T}_{\mathrm{C}}\right)}\right|\varDelta \left({T}_{\mathrm{H}}-{T}_{\mathrm{C}}\right)=\\ {}\frac{\varDelta {Q}_{net}}{Q_{net}}+\frac{\varDelta A}{A}+\frac{\varDelta \left({T}_{\mathrm{H}}-{T}_{\mathrm{C}}\right)}{\left({T}_{\mathrm{H}}-{T}_{\mathrm{C}}\right)}\end{array} $$

The error transfer formula of Nusselt number is as follows [19]:

12 $$ \begin{array}{l}\frac{\varDelta \mathrm{Nu}}{\mathrm{Nu}}=\left|\frac{\partial \mathrm{lnNu}}{\partial h}\right|\varDelta h+\left|\frac{\partial \mathrm{lnNu}}{\partial W}\right|\varDelta W+\left|\frac{\partial \mathrm{lnNu}}{\partial {\lambda}_{\mathrm{f}}}\right|\varDelta {\lambda}_{\mathrm{f}}=\\ {}\frac{\varDelta h}{h}+\frac{\varDelta W}{W}+\frac{\varDelta {\lambda}_{\mathrm{f}}}{\lambda_{\mathrm{f}}}\end{array} $$

Based on the Eqs. (10) and (11), the errors of the convective heat transfer coefficient and Nusselt number are 5.65 and 6.34%, respectively, in this experiment. It can be found that the errors of the experimental sets are small, which can ensure the reliability and accuracy of the experimental results.

## Results and Discussions

### Experiment Validation

**Fig. 7:**
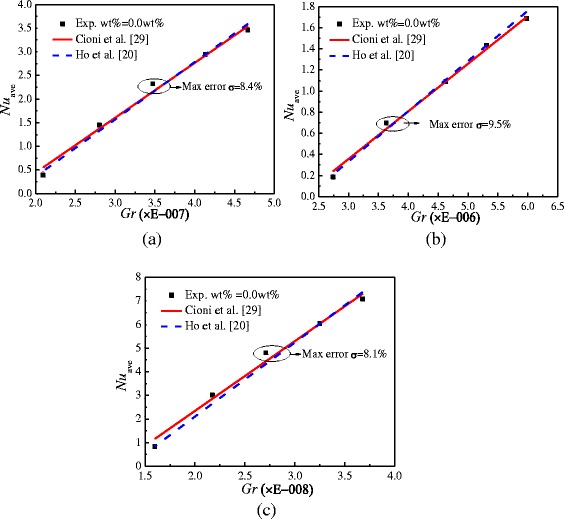
Experiment set validation. Comparison of Nusselt numbers between the experimental results and the published literatures in enclosures with two different aspect ratios. **a** *A* = 1:2. **b** *A* = 1:4. **c** *A* = 1:1

Before the study on nanofluid, the experiment validation is necessary. Figure 7 shows the comparison of Nusselt numbers between the experimental results of water and the results of published literatures for enclosures with *A* = 1:2, *A* = 1:4, and *A* = 1:1. The max errors for enclosures with *A* = 1:2, *A* = 1:4, and *A* = 1:1 are 8.4, 9.5, and 8.1%, respectively. It can be found that the experimental results have a good agreement with the results of published literatures [20, 29], which verifies the accuracy and reliability of the experimental system.

### Enclosure with *A* = 1:2

**Fig. 8:**
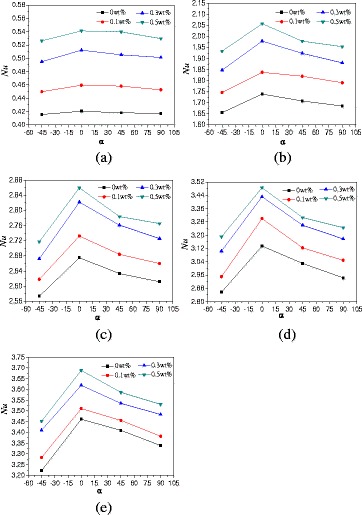
Changes of Nusselt numbers with rotation angles (*A* = 1:2). Average Nusselt numbers change of nanofluid with rotation angles of enclosure (*A* = 1:2) at different heating powers. **a** *Q* = 1 W. **b** *Q* = 5 W. **c** *Q* = 10 W. **d** *Q* = 15 W. **e** *Q* = 20 W

The effects of rotation angles on the natural convection heat transfer characteristics of TiO_2_-water nanofluid are discussed in this paper. Figure 8 presents the changes of average Nusselt numbers with the rotation angles of enclosure with *A* = 1:2. It can be found from Fig. 8 that Nusselt numbers firstly increase and then decrease with the rotation angles. The enclosure with rotation angle *α* = 0° has the highest Nusselt number followed by the enclosure with rotation angles *α* = 45° and *α* = 90°, the enclosure with rotation angle *α* = −45° has the lowest Nusselt number. Heat conduction becomes playing more and more role when the rotation angle decreases (*α* ≤ −90°), and the heat transfer is almost heat conduction when the rotation angle decreases to *α* = −90°. When the hot wall locates in the top and the cold wall locates in the bottom of enclosure (*α* = −90°), the direction of buoyancy is upward, but the top wall prevents the fluid moving upward. The movement of nanofluid in the enclosure is small, and the main heat transfer is the heat conduction, which causes a small Nusselt number. The enclosure with *α* = −45° is more close to the enclosure with *α* = −90° and shows the smallest Nusselt number compared with other rotation angles. For the enclosures with rotation angles *α* = 45° and *α* = 90°, the fluid near the bottom hot wall is heated and moves upward and the fluid near the top cold wall is cooled and moves downward. The directions of hot fluid and cold fluid are opposite and prevent the natural convection heat transfer, which cause a lower Nusselt number compared with the enclosure with *α* = 0° but a higher Nusselt number compared with the enclosure with *α* = −45°. It can be also seen that the differences between various rotation angles increases with the heating power. This is because the effects of rotation angles play the main role on heat transfer at low heating power, and the effects of convective on heat transfer are small. However, the convective heat transfer intensity increases with the heating power and plays the main role on heat transfer at high heating power, which causes the bigger differences between the various rotation angles at high heating power compared with that at low heating power.

**Fig. 9:**
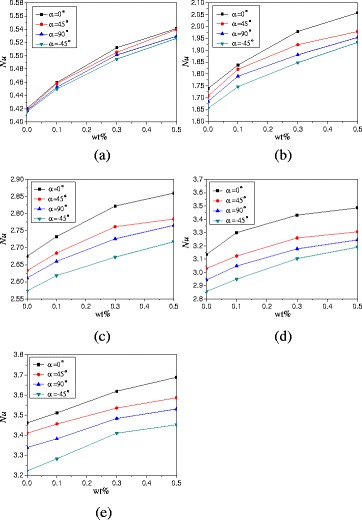
Changes of Nusselt numbers with nanoparticle mass fractions (*A* = 1:2). Average Nusselt numbers changes of nanofluid in the enclosure (*A* = 1:2) with nanoparticle mass fractions at different heating powers. **a** *Q* = 1 W. **b** *Q* = 5 W. **c** *Q* = 10 W. **d** *Q* = 15 W. **e** *Q* = 20 W

In addition to the rotation angles, the effects of nanoparticle mass fraction on the natural convection heat transfer are also discussed. Figure 9 shows the changes of average Nusselt numbers with nanoparticle mass fractions. It can be found that Nusselt numbers increase with nanoparticle mass fractions. For heating power *Q* = 1 W and *α* = 0°, TiO_2_-water nanofluid with wt% = 0.1%, wt% = 0.3%, and wt% = 0.5% can enhance the heat transfer by 9.3, 21.8, and 28.7% compared with water, respectively. The enhancement ratio decreases with the heating power. For heating power *Q* = 20 W and *α* = 0°, TiO_2_-water nanofluid with wt% = 0.1%, wt% = 0.3%, and wt% = 0.5% can enhance the heat transfer by 1.4, 4.6, and 6.6% compared with water, respectively. The turbulence intensity becomes playing a major role at high heating power, and the effects of nanoparticle mass fraction on heat transfer become small.

**Fig. 10:**
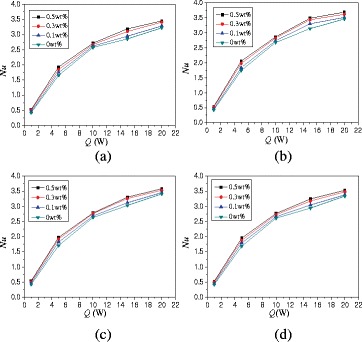
Changes of Nusselt numbers with heating power (*A* = 1:2). Average Nusselt numbers changes of nanofluid in the enclosure (*A* = 1:2) with heating power at different rotation angles. **a** α = −45°. **b** α = 0°. **c** α = 45°. **d** α = 90°

The effects of heating powers on the natural convection heat transfer are studied in this paper. Figure 10 shows the changes of average Nusselt numbers with heating power. For *α* = 0°, TiO_2_-water nanofluid at *Q* = 5 W, *Q* = 10 W, *Q* = 15 W, and *Q* = 20 W can enhance the heat transfer by 280.2, 428.4, 544.1, and 581.5% compared with that at *Q* = 1 W. High heating power enhances the turbulence intensity and improves the heat transfer.

### Enclosure with *A* = 1:4

**Fig. 11:**
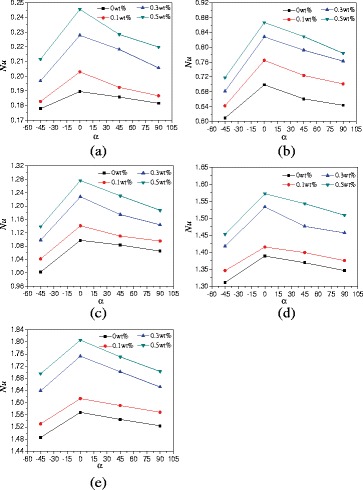
Changes of Nusselt numbers with rotation angles (*A* = 1:4). Average Nusselt numbers changes of nanofluid with rotation angles of enclosure (*A* = 1:4) at different heating powers. **a** *Q* = 1 W. **b** *Q* = 5 W. **c** *Q* = 10 W. **d** *Q* = 15 W. **e** *Q* = 20 W

In order to investigate the effects of aspect ratios of enclosures on the heat transfer, the natural convection heat transfer characteristics of enclosure with *A* = 1:4 filled with TiO_2_-water nanofluid are studied. Figure 11 gives the changes of average Nusselt numbers with the rotation angles of enclosure. It can be obtained that a similar conclusion like *A* = 1:2 that Nusselt numbers firstly increase and then decrease with the rotation angles. For nanofluid with wt% = 0.5% example, the differences between *A* = 1:4 and *A* = 1:2 are that the enhancement ratios (from 6.5 to 20.7%) of Nusselt number in the enclosure (*A* = 1:4, *α* = 0°) compared with that in the enclosure (*A* = 1:4, *α* = −45°) are higher than the enhancement ratios (from 2.85 to 9.3%) of Nusselt number in the enclosure (*A* = 1:2, *α* = 0°) compared with that in the enclosure (*A* = 1:2, *α* = −45°).

**Fig. 12:**
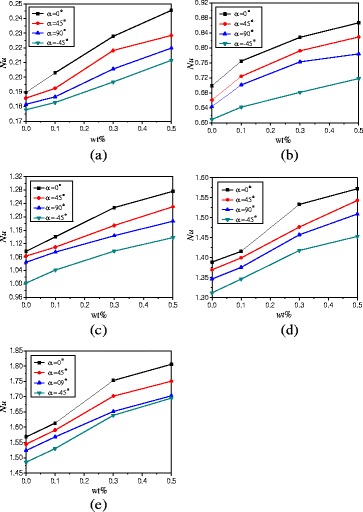
Changes of Nusselt numbers with nanoparticle mass fractions (*A* = 1:4). Average Nusselt numbers changes of nanofluid in the enclosure (*A* = 1:4) with nanoparticle mass fractions at different heating powers. **a** *Q* = 1 W. **b** *Q* = 5 W. **c** *Q* = 10 W. **d** *Q* = 15 W. **e** *Q* = 20 W

Figure 12 presents the changes of average Nusselt numbers with nanoparticle mass fractions. For heating power *Q* = 1 W and *α* = 0°, TiO_2_-water nanofluid with wt% = 0.1%, wt% = 0.3%, and wt% = 0.5% can enhance the heat transfer by 7.1, 20.2, and 29.5% compared with water, respectively. The enhancement ratio decreases with the heating power. For heating power *Q* = 20 W and *α* = 0°, TiO_2_-water nanofluid with wt% = 0.1%, wt% = 0.3%, and wt% = 0.5% can enhance the heat transfer by 2.9, 11.8, and 15.1% compared with water, respectively.

**Fig. 13:**
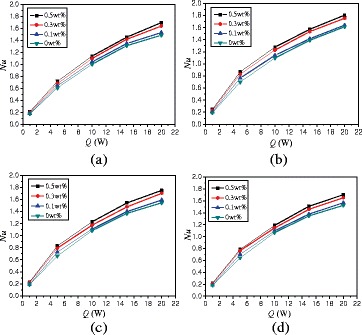
Changes of Nusselt numbers with heating power (*A* = 1:4). Average Nusselt numbers changes of nanofluid in the enclosure (*A* = 1:4) with heating power at different rotation angles. **a** α = −45°. **b** α = 0°. **c** α = 45°. **d** α = 90°

Figure 13 shows the changes of average Nusselt numbers with heating power. Average Nusselt numbers of nanofluid can be enhanced by 242.4% ~ 701.5% compared with water at heating power Q = 1 W. For *α* = 0°, TiO_2_-water nanofluid with wt% = 0.5% at *Q* = 5 W, *Q* = 10 W, *Q* = 15 W, and *Q* = 20 W can enhance the heat transfer by 253.0, 419.9, 540.3, and 635.6% compared with that at *Q* = 1 W, respectively.

### Comparison Between *A* = 1:2, *A* = 1:4, and *A* = 1:1

**Fig. 14:**
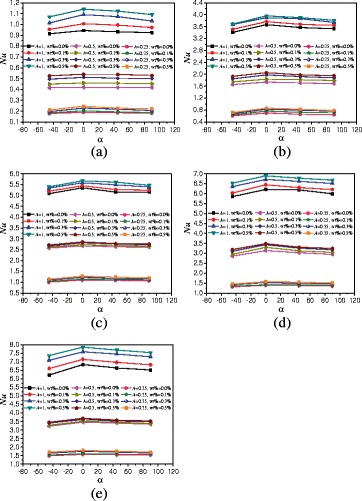
Nusselt numbers comparison between different aspect ratios. Comparison of average Nusselt numbers of nanofluid in different aspect ratios (*A* = 1:1, *A* = 1:2, and *A* = 1:4) and rotation angle enclosures at different heating powers. **a** *Q* = 1 W. **b** *Q* = 5 W. **c** *Q* = 10 W. **d** *Q* = 15 W. **e** *Q* = 20 W

Due to the length limitation of this paper, the results of enclosure with *A* = 1:1 are only given in Fig. 14, and the effects of different rotation angles, nanoparticle mass fractions, and heating powers on heat transfer can all be shown in Fig. 14. In order to compare the heat transfer characteristics of enclosures with *A* = 1:2, *A* = 1:4, and *A* = 1:1, Fig. 14 shows the comparison of average Nusselt numbers between *A* = 1:2, *A* = 1:4, and *A* = 1:1 at different rotation angles. It is found that the Nusselt numbers increase with the aspect ratio of enclosure. The Nusselt numbers of enclosure (*A* = 1:1 and *A* = 1:2) can be enhanced by 190.6% ~ 224.4% and 103.6% ~ 172.0% compared with the Nusselt numbers of enclosure (*A* = 1:4) at the same conditions, respectively. For *Q* = 1 W and *α* = 0° example, nanofluid with wt% = 0.5%, wt% = 0.3%, wt% = 0.1%, and wt% = 0.0% in the enclosure with *A* = 1:2 can enhance the heat transfer by 120.4, 124.9, 126.5, and 121.9% compared with that in the enclosure with *A* = 1:4. The enhancement ratio decreases with the heating power. vFor *Q* = 20 W and *α* = 0°, nanofluid with wt% = 0.5%, wt% = 0.3%, wt% = 0.1%, and wt% = 0.0% in the enclosure with *A* = 1:2 can enhance the heat transfer by 104.2, 106.5, 117.6, 120.7% compared with that in the enclosure with A = 1:4. It is also found that Nusselt number increases from wt% = 0.1% to wt% = 0.3% are bigger than that from wt% = 0.3% to wt% = 0.5%. This is because the increase of thermal conductivity plays the main role in the heat transfer from wt% = 0.1% to wt% = 0.3%, which causes a big enhancement. But the increase of viscosity begins to play the main role in the heat transfer from wt% = 0.3% to wt% = 0.5%, which causes a small enhancement. Because Fig. 14 can cover all the experimental results, the detailed results of Fig. 14 are shown in Tables 2, 3, and 4.

**Table 2:** Nusselt numbers (*A* = 1:1). Nusselt number values based on Fig. 14 (*A* = 1:1)

*Q*	α	Nu (0.5%)	Nu (0.3%)	Nu (0.1%)	Nu (0%)
1 W	−45	1.07043	1.01428	0.95476	0.91431
	0	1.14055	1.09147	1.00692	0.94354
	45	1.12296	1.07193	0.99404	0.93241
	90	1.09139	1.0319	0.97131	0.92421
5 W	−45	3.68552	3.66999	3.50965	3.40783
	0	3.96164	3.90255	3.76721	3.66701
	45	3.90241	3.86575	3.67688	3.56889
	90	3.81346	3.74198	3.64248	3.52605
10 W	−45	5.40131	5.3391	5.19836	5.08178
	0	5.67641	5.58409	5.44825	5.35755
	45	5.61314	5.49212	5.27315	5.15387
	90	5.47057	5.39303	5.24816	5.16605
15 W	−45	6.53082	6.35713	6.03166	5.85206
	0	6.89679	6.71681	6.44823	6.21186
	45	6.7772	6.62197	6.30624	6.18767
	90	6.68041	6.51411	6.20766	5.99525
20 W	−45	7.36842	7.09355	6.61076	6.22726
	0	7.86642	7.59036	7.1488	6.84292
	45	7.69319	7.45785	6.97388	6.64521
	90	7.54729	7.3013	6.82694	6.52435

**Table 3:** Nusselt numbers (*A* = 1:2). Nusselt number values based on Fig. 14 (*A* = 1:2)

*Q*	α	Nu(0.5%)	Nu(0.3%)	Nu(0.1%)	Nu(0%)
1 W	−45	0.21153	0.19679	0.18278	0.17788
	0	0.24552	0.22783	0.20294	0.18955
	45	0.2284	0.21822	0.19236	0.18577
	90	0.21981	0.20564	0.18664	0.18157
5 W	−45	0.71806	0.68137	0.64216	0.6091
	0	0.86677	0.82804	0.76474	0.69884
	45	0.829	0.79205	0.72391	0.66098
	90	0.78393	0.76264	0.70126	0.64364
10 W	−45	1.13829	1.09805	1.04143	1.00264
	0	1.27625	1.22757	1.14081	1.09706
	45	1.23027	1.17417	1.10987	1.08314
	90	1.18711	1.14366	1.09506	1.06465
15 W	−45	1.45308	1.41801	1.3465	1.31214
	0	1.572	1.53298	1.41562	1.38905
	45	1.54297	1.47638	1.39931	1.36964
	90	1.50899	1.45712	1.37573	1.34674
20 W	−45	1.69537	1.63891	1.53034	1.48585
	0	1.80587	1.75282	1.61349	1.56828
	45	1.75054	1.70163	1.59055	1.54486
	90	1.70272	1.65153	1.56853	1.52402

**Table 4:** Nusselt numbers (*A* = 1:4). Nusselt number values based on Fig. 14 (*A* = 1:4)

*Q*	α	Nu(0.5%)	Nu(0.3%)	Nu(0.1%)	Nu(0%)
1 W	−45	0.5263	0.495	0.44995	0.4157
	0	0.54119	0.51222	0.45962	0.42056
	45	0.53964	0.50517	0.45825	0.4182
	90	0.52953	0.5012	0.45265	0.41729
5 W	−45	1.93363	1.84772	1.74651	1.6555
	0	2.05786	1.97908	1.83774	1.73927
	45	1.97887	1.9236	1.82002	1.70807
	90	1.95401	1.88076	1.79071	1.68525
10 W	−45	2.71752	2.67225	2.61801	2.57357
	0	2.85976	2.82194	2.73248	2.67519
	45	2.78396	2.76133	2.6841	2.63338
	90	2.76545	2.72596	2.65979	2.61159
15 W	−45	3.19016	3.1043	2.94978	2.85708
	0	3.4856	3.43158	3.2998	3.13513
	45	3.3054	3.25965	3.12342	3.03013
	90	3.24525	3.17768	3.0485	2.94331
20 W	−45	3.45233	3.41039	3.28328	3.22254
	0	3.68838	3.61935	3.51132	3.46162
	45	3.58654	3.53537	3.45595	3.40981
	90	3.53074	3.48353	3.38208	3.33931

## Conclusions

The stability and natural convection heat transfer characteristics of the two enclosures with different rotation angles (*α* = −45°, *α* = 0°, *α* = 45°, and *α* = 90°) filled with TiO_2_-water nanofluid are experimentally investigated. Some conclusions are obtained as follows:

1. TiO_2_-water nanofluid with *m* = 6 wt% and pH = 8 has the lowest transmittance and has the best stability.
2. The enclosure with rotation angle *α* = 0° has the highest Nusselt number followed by the enclosure with rotation angles *α* = 45° and *α* = 90°; the enclosure with rotation angle *α* = −45° has the lowest Nusselt number.
3. There is a higher heat transfer performance in a bigger aspect ratio enclosure. The Nusselt numbers of enclosure (*A* = 1:1 and *A* = 1:2) can be enhanced by 190.6% ~ 224.4% and 103.6% ~ 172.0% compared with the Nusselt numbers of enclosure (*A* = 1:4) at the same conditions.
4. Nusselt numbers increase with nanoparticle mass fractions, but the enhancement ratio decreases with the heating power.
5. Average Nusselt numbers increase with the heating power. Average Nusselt numbers of nanofluid can be enhanced by 701.5% compared with water at the best.
